# The environmental impact of community caries prevention - part 1: fluoride varnish application

**DOI:** 10.1038/s41415-022-4901-7

**Published:** 2022-08-26

**Authors:** Alexandra Lyne, Paul Ashley, Mark Johnstone, Brett Duane

**Affiliations:** 4141510381001 Paediatric Dentistry, Eastman Dental Institute, Rockefeller, 21 University Street, London, WC1E 6DE, UK; 4141510381002 Kent Community Health NHS Foundation Trust, Capital House, Jubilee Way, Faversham, Kent, ME13 8GD, UK; 4141510381003 Dental Public Health, Dublin Dental Hospital, Trinity College Dublin, D02 F859, Ireland

## Abstract

**Background** Healthcare is a significant contributor to climate change and planetary health. Prevention of oral disease, such as caries, is an important part of any mechanism to improve sustainability. Caries prevention includes community schemes such as water fluoridation, toothbrushing, or fluoride varnish (FV) application. The aim of this study was to quantify the environmental impact of FV application.

**Materials and methods** A comparative life cycle assessment (LCA) was conducted to quantify the environmental impact of a five-year-old child receiving two FV applications in a one-year period in schools and in dental practice.

**Results** FV application in dental practice during an existing appointment had the lowest environmental impact in all 16 categories, followed by FV application in schools. FV application at a separate dental practice appointment had the highest impact in all categories, with a majority of the impact resulting from the patient travel into dental practice.

**Discussion** FV application while a child is already attending dental practice (for example, at routine recall) is the most sustainable way to deliver FV. School FV programmes are an alternative, equitable way to reach all children who may not access routine care in dental practice.

**Supplementary Information:**

Zusatzmaterial online: Zu diesem Beitrag sind unter 10.1038/s41415-022-4901-7 für autorisierte Leser zusätzliche Dateien abrufbar.

## Key points

- This paper is the first in a three-part series looking at the environmental impact of different community-level caries prevention programmes.
- This paper quantifies the environmental impact of fluoride varnish application in children.
- This paper suggests how fluoride varnish application might be made more sustainable.

**Supplementary Information:**

Zusatzmaterial online: Zu diesem Beitrag sind unter 10.1038/s41415-022-4901-7 für autorisierte Leser zusätzliche Dateien abrufbar.

## Introduction

It is now recognised that the world is in significant danger from environmental factors. It faces rapidly rising carbon dioxide levels, reductions in worldwide biodiversity, increased air pollution and increased water eutrophication. The list of deterioration continues and can be found in the sixth Intergovernmental Panel on Climate Change report.^1^

The health sector consumes a considerable volume of resources and generates significant amounts of waste. The NHS is responsible for approximately 4% of the UK's total emissions.^2^ Dentistry on its own is responsible for the production of around 675,000 kilotonnes of carbon equivalent emissions per year (England, 2014-2015 figures).^3^ The NHS has now committed to a net zero emission strategy.^2^

There are many routes the NHS can take to make healthcare more sustainable, ranging from better use of estates, reducing travel and minimising waste.^4^^,^^5^^,^^6^ Like most complex environmental or public health problems, a multifaceted approach is needed to improve sustainability.^7^ Preventive approaches are an important part of making healthcare more sustainable, with an additional wide range of economic and social benefits.^8^

Preventive approaches in dentistry are focused primarily on dental caries as it is the world's most chronic disease, affecting more than 3.5 billion people.^9^ There are a range of preventative therapies available that can be delivered at population or individual level. Using community-wide preventive interventions appropriately will reduce the economic, ethical and environmental burden of the clinical dental management of established caries disease.

The role of fluoride in the prevention of dental caries is well established. Public Health England recommends four evidence-based and community-based caries prevention programmes for children: water fluoridation, the application of fluoride varnish, supervised toothbrushing and the targeted provision of toothbrushes and toothpaste to populations in need.^10^^,^^11^^,^^12^ Clinical-effectiveness and cost-effectiveness data have been combined to produce a return on investment tool, for example, commissioning a fluoride varnish in-school programme had a return of investment over ten years of £2.74 for every pound spent.^13^ This document is a driver for procurement and commissioning decisions in the UK.

To date, there are no environmental impact studies for these different community methods for preventing dental caries. This lack of clarity makes it difficult for commissioners of preventive services to take environmental sustainability into account when making decisions about what preventative programmes to fund.

Life cycle assessment (LCAs) is used both in industry and in healthcare to understand the environmental impacts of a particular service or product. In healthcare, LCA studies have been undertaken in the field of anaesthetics.^14^ In dentistry, LCA has been used to measure the impact of a dental examination and products such as toothbrushes.^15^^,^^16^ LCAs are arguably more comprehensive than just a carbon footprint alone as they consider multiple measures of environmental sustainability and not just climate change. LCA data can also be converted into disability adjusted life years (DALYs) to quantify the human health burden of a service or produce. The European Union and International Standardisation Organisation (ISO) set guidance to aid consistent and transparent LCA reporting.^17^^,^^18^

In order to choose the most appropriate preventive programme, it is important that decision-makers in healthcare look at the 'triple bottom line' - clinical effectiveness (for example, systematic reviews), cost effectiveness (for example, return on investment tools) and environmental sustainability (for example, LCA). In this series of three papers, the environmental impact each of these preventive programmes is quantified, in order to identify the most sustainable approach.

The aim of this first paper was to quantify the environmental impacts of fluoride varnish (FV) application in children using LCA methodology. The objective was to model a community-delivered FV application programme in schools and compare the results to FV application in dental practice.

## Materials and methods

The primary outcome measure was the life cycle impact assessment (LCIA) and secondary outcome measures included normalised results, contribution analysis, and DALYs. The LCA was undertaken at Dublin Dental University Hospital (Trinity College Dublin) in partnership with the Eastman Dental Hospital, London.

**Fig. 1:**
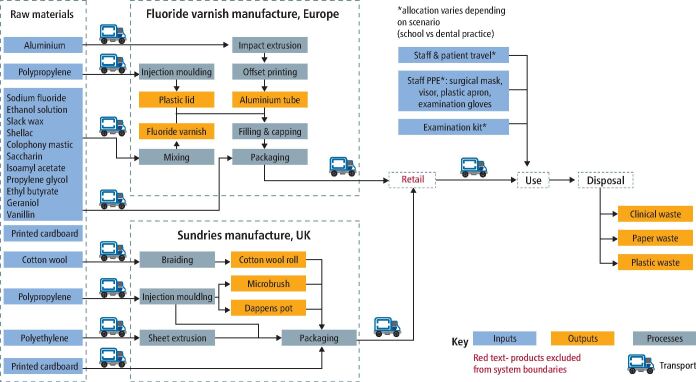
System boundaries for FV application

To align with Public Health England's recommended prevention schemes to prevent dental caries in five-year-old children, the functional unit was defined as an individual five-year-old child receiving FV application twice over a one-year period.^10^ The system boundaries are shown in Figure 1. Three scenarios were compared:

- The child receives FV application in their school, delivered by a community dental service
- The child receives FV application in dental practice, during an existing appointment (for example, routine recall)
- The child receives FV in dental practice, at a separate appointment (that is, child attending the practice solely for FV).

LCA methodology was applied in line with ISO standards (ISO 2015) and European Union Product Environmental Footprint (PEF) 2019 guidance.^17^^,^^18^ In total, 16 impact categories were examined in this study and the LCIA methods were based on PEF guidance and are described in Table 1. The software OpenLCA v1.11 was used alongside the reference database Ecoinvent v3.7.1 for the LCIA and ReCiPe (2016) Endpoint (H) was used to calculate DALYs. The LCIA results were normalised against per capita reference values and contribution analysis carried out.

**Table 1:** Impact categories and LCIA methods

Impact category (abbreviation)	LCIA method (units)	Description
Climate change (CC)	IPCC 2013 GWP 100a (kg CO2 eq)	Potential for global warming from greenhouse gas emissions
Ecosystem quality: freshwater and terrestrial acidification (EAC)	ILCD 2011 Midpoint+ (Mol H+ eq)	Acidification of soils and freshwater due to gas release
Ecosystem quality: ecotoxicity freshwater (ECF)	ILCD 2011 Midpoint+ (CTUe)	Harmful effects of toxic substances on freshwater organisms
Ecosystem quality: eutrophication freshwater (EUF)	ILCD 2011 Midpoint+ (kg P eq)	Changes in freshwater organisms and ecosystems caused by excess nutrients
Ecosystem quality: eutrophication marine (EUM)	ILCD 2011 Midpoint + (kg N eq)	Changes in marine organisms and ecosystems caused by excess nutrients
Ecosystem quality: eutrophication terrestrial (EUT)	ILCD 2011 Midpoint + (Molc N eq)	Changes in land organisms from excess nutrients in soil and air
Human health: cancer effects (HCE)	ILCD 2011 Midpoint+ (CTUh)	Harm to human health that causes or increases cancer risk
Human health: ionizing radiation (HIR)	ILCD 2011 Midpoint + (kBq U-235 eq)	Potential damage to human DNA from ionizing radiation
Human Health: non-cancer effects (HNC)	ILCD 2011 Midpoint+ (CTUh)	Harm to human health that is not related to cancer or ionising radiation
Human health: respiratory inorganics (HRI)	PM method (Disease inc)	Harm to human health caused by particulate matter emissions (respiratory disease)
Human health: photochemical ozone formation (HOF)	ILCD 2011 Midpoint + (kg NMVOC eq)	Harm to human health from gas emissions that contribute to smog in the lower atmosphere
Resource use: land use (RLU)	Soil quality index based on LANCA (Pt)	Depletion of natural resources, change in soil quality, and reduction in biodiversity
Human health: ozone depletion (HOD)	ILCD 2011 Midpoint+ (kg CFC11 eq)	Air emissions causing stratospheric ozone layer destruction
Resource use: fossils (RFF)	CML-IA baseline (MJ)	Depletion of natural fossil fuels
Resource use: minerals and metals (RMM)	CML-IA baseline (kg Sb eq)	Depletion of natural non-fossil fuel resources
Resource use: dissipated water (RDW)	AWARE (m3 depriv)	Potential for water deprivation to humans and ecosystems globally

A life cycle inventory was created for each scenario, based on the assumptions described below, as seen in the online Supplementary Information.

### Assumptions for fluoride varnish

Based on manufacturer's recommendations for a five-year-old, it was assumed that 0.25 ml of FV was used per application and that there was no wastage of varnish product. Therefore, one tube of fluoride varnish can be used for 40 applications. It was assumed the child would have two applications per year, based on UK clinical guidance.^10^

The FV was based on a product readily available on the UK market, although the brand has been anonymised. The varnish was supplied in a 10 ml tube, packaged in a cardboard box. The ingredients of the varnish were based on the information leaflet provided by the manufacturer. For ingredients that did not have a specified amount, it was assumed the ingredients were equal in weight. Two of the flavouring ingredients (iris resinoid and jasmine absolute) were not available in the Ecoinvent database and where therefore excluded. The manufacturing process was estimated in kWh, based on a 3 kW mixing machine that is used for toothpaste and medicines, with a two-hour mixing programme and 2,000 L barrel. The mixed FV was packaged in a printed and sealed aluminium tube with a polypropylene lid, contained in a cardboard box. The weight of the packaging was determined from a tear down of a sample product. The packaging processes of filling, sealing and screw capping the tubes were estimated from the kWh of the machinery, based on a 5 kW tube filling and sealing machine and a 15 kW screw capping machine.

The transport of the packaged varnish from the manufacturing location in mainland Europe to the UK was assumed to be via lorry and ferry to the population centre of the UK. Distances were assumed from the shortest available route on Google Maps (2021). Once empty, it was assumed the tube was disposed of in clinical waste and the cardboard box in recycling.

### Assumptions on sundries needed for fluoride varnish application

Sundries referred to all the instruments, disposable equipment and personal protective equipment (PPE) needed for fluoride varnish application. Pre-existing LCA data for some of these sundries were available from projects involving the authors at Trinity College Dublin (BD, AL), including the disposable examination kit,^19^ surgical mask and visor^20^ and examination gloves.^21^ The remaining sundries (cotton rolls, dappens, microbrush, pulp tray, plastic apron) and their packaging were modelled from a teardown of sample products. They were all assumed to be manufactured in the UK (the geographical location of a known production factory in North West England was used) and transported via lorry and ferry from the factory to the UK population centre (just outside of Derby, England).^22^ All sundries were assumed to be disposed of in clinical waste and the packaging recycled. The exact sundries for application of fluoride varnish in school and in dental practice are described in Table 2.

**Table 2:** Sundries needed for FV application in schools and dental practice

Item	Number needed for one application of fluoride varnish		
	In school	In practice, at FV only appointment	In practice, during an existing appointment
Cotton wool roll	2	2	2
Dappens pot	1	1	1
Microbrush	1	1	1
Pulp tray	1	0	0
Examination kit (mirror, probe, tweezers)	1 (disposable kit)	1 (reusable kit)	0 (assumed kit already open for existing appointment)
PPE (apron, mask, visor, pair of gloves)	1	1	0 (assumed kit already open for existing appointment)

Energy use to keep the school and dental practice open was excluded from the system boundaries. The cleaning of the dental chair or school chair was also excluded from all scenarios.

### Assumptions on staff and patient travel

Based on a study by Public Health England, it was assumed that dental staff commute an average of 21 miles each way to and from their place of work and patients travel an average of 7.57 miles for a return journey to their local dental practice.^23^ The method of travel for patients and staff was based on data on commuting journeys from the Department of Transport, which estimated that approximately 67% travel by car, 9% by bus, 5% by train, 4% by bicycle and 11% by foot.^24^

In order to provide FV application in school, it was assumed that two dental care professionals travelled to and from their place of work (community dental centre). In consultation with an existing FV programme, it was assumed that two staff members drove a small van (Euro 5 engine) on an 11 km round trip to the school, which was the average distance of the schools in their programme. It was assumed an average of 90 children were treated in a day, which took up the entire working day for the dental staff. There was no patient travel allocated as the children were already in school.

For FV application at an existing dental practice appointment, two minutes out of a 7.5-hour working day were allocated (0.42% of daily staff travel) for two members of staff - a dentist and a dental assistant. There was no patient travel allocated as the children were already attending the practice.

For FV application at a separate dental practice appointment, five minutes out of the working day were allocated (1.04% of daily staff travel) for one member of staff - a dentist or dental care professional (for example, a dental assistant with additional training in FV application or a dental therapist). As the child was travelling to the practice specifically for FV application, one round trip patient journey was allocated. It was assumed the five-year-old child was brought to the appointment by one parent or carer and they travelled together, to and from home.

## Results

The results of the LCIA are shown in Table 3. The greatest impact in all 16 categories came from applying FV at a separate practice appointment. For climate change potential, applying fluoride at a separate practice visit produced the equivalent of 8.12 kg of carbon dioxide compared to 3.31 kg for applying the varnish in schools and just 1.09 kg applying the varnish when the child is already attending practice for another appointment.

**Table 3:** LCIA results for FV application

Impact category (units)	In school	During existing practice appointment	At separate practice appointment
Climate change (kg CO2 eq)	3.31E+00	1.09E+00	8.12E+00
Acidification (mol H+ eq)	1.14E-02	2.51E-03	3.17E-02
Freshwater ecotoxicity (CTU)	7.04E+00	2.09E+00	1.85E+01
Freshwater eutrophication (kg P eq)	6.70E-04	2.10E-04	1.41E-03
Marine eutrophication (kg N eq)	3.18E-03	1.27E-03	8.35E-03
Terrestrial eutrophication (mol N eq)	2.66E-02	6.94E-03	8.25E-02
Carcinogenic effects (ctuh)	2.99E-07	3.98E-08	3.64E-07
Ionising radiation (kg U235 eq)	1.40E-01	3.92E-02	4.99E-01
Non-carcinogenic effects (ctuh)	3.35E-07	7.33E-08	7.15E-07
Ozone layer depletion (kg CFC-11 eq)	3.12E-07	1.13E-07	1.23E-06
Photochemical ozone creation (kg NMVOC eq)	8.14E-03	1.92E-03	2.72E-02
Respiratory inorganics effects (disease inc)	1.32E-07	2.45E-08	3.27E-07
Dissipated water (m3 water eq)	1.22E+00	7.17E-01	1.75E+00
Fossil use (MJ)	3.57E+01	7.52E+00	1.07E+02
Land use (pts)	2.01E+01	5.88E+00	6.15E+01
Mineral/metal use (kg Sb eq)	2.64E-05	4.95E-06	8.92E-05

**Fig. 2:**
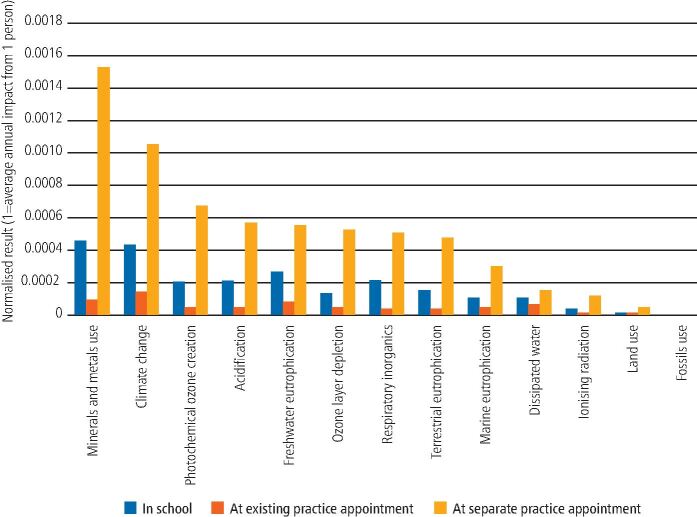
Normalised results for FV application

The LCIA results were normalised against average global reference values for the annual environmental footprint of the average person, as shown in Figure 2. Following PEF recommendations, the three toxicity-related categories have been excluded while the robustness of the methodology is under review.^17^ Mineral and metal use and climate change had the greatest normalised impacts in all scenarios.

**Fig. 3:**
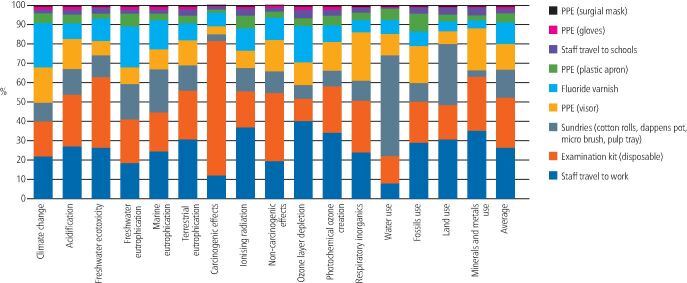
Contribution analysis for FV application in school

**Fig. 4:**
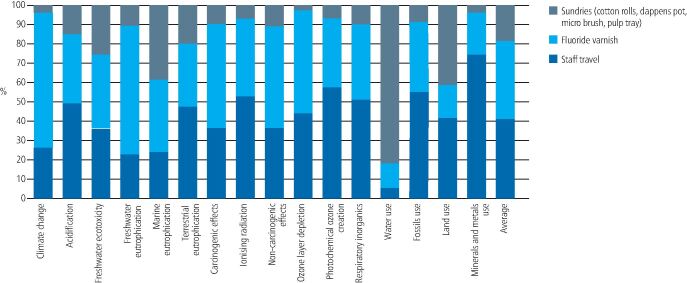
Contribution analysis for FV application during an existing dental practice appointment

**Fig. 5:**
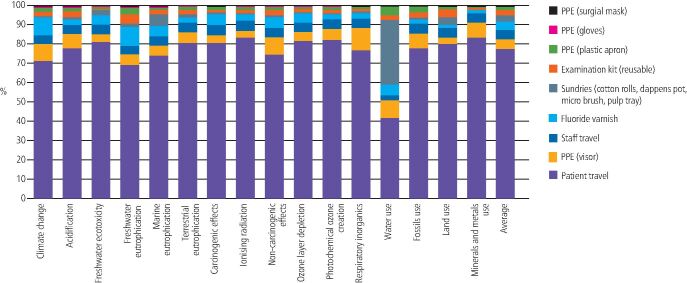
Contribution analysis for FV application at a separate dental practice appointment

Figures 3, 4 and 5 shows the contribution analysis for each impact category. For applying fluoride varnish in schools, the biggest contributors were staff travel (this was two members of staff travelling from their home to their place of work, that is, a community dental clinic) and the disposable examination kit. Staff travel contributed an average of 26.22% of the impact result (range 7.71-40.33%) and the examination kit contributed an average of 26.02% (range 11.46-69.51%. The FV itself only contributed an average of 10.96% to the overall impact (range 4.01-22.69%).

For FV application during an existing practice appointment, there were only three contributing factors, which was the FV itself (contributing an average of 40.07%), the allocation of staff travel (contributing an average of 41.1%) and the extra sundries needed to apply the varnish (contributing an average of 18.83%). There was no examination kit, staff PPE, or patient travel allocated in this scenario, as it was assumed the patient was already sitting in the dental chair with instruments open and staff already in PPE.

For FV application at a separate practice appointment, it was the patient travel that had the greatest contribution (average 77.60%). All other factors contributed an average of 5% or less to the overall impact. The FV itself contributed an average of 4.39%.

Table 4 shows the DALY calculations. FV in schools produced 125 seconds worth of DALYs compared to 49 seconds for the existing practice appointment and 279 seconds for the separate practice appointment. For all scenarios, climate change was the biggest contributor the overall DALY result (66-86%), followed by water consumption (14-34%). All other human health categories contributed less than 0.01% to the overall DALY result.

**Table 4:** DALYs for FV application

Human health impact category	In school	During existing practice appointment	At separate practice appointment
Global warming	3.07136E-06	1.00952E-06	7.53245E-06
Stratospheric ozone depletion	1.65683E-10	6.00349E-11	6.53135E-10
Ionizing radiation	1.18975E-09	3.3286E-10	4.24116E-09
Respiratory inorganics	8.33477E-11	1.54435E-11	2.06061E-10
Photochemical ozone formation	7.13287E-09	1.68245E-09	2.37996E-08
Cancerous effects	9.9197E-13	1.32268E-13	1.20925E-12
Non-cancerous effects	2.22814E-15	4.8772E-16	4.75326E-15
Water consumption	8.97221E-07	5.27426E-07	1.28566E-06
Total DALYs	3.97716E-06	1.53903E-06	8.84701E-06
DALY seconds (rounded)	125	49	279

A sensitivity analysis was performed for fluoride varnish application in schools, swapping the disposable examination kits for reusable examination kits. This resulted in a lower impact in all categories, as shown in Table 5. Climate change impact reduced by 0.42 kg (13%), mineral and metal use by 25% and photochemical ozone formation by 18%.

**Table 5:** Sensitivity analysis of disposable vs reusable examination kits for FV in schools

Impact category (units)	Fluoride varnish application in schools	
	Disposable exam kit	Reusable exam kit
Climate change (kg CO2 eq)	3.31E+00	2.89E+00
Acidification (mol H+ eq)	1.14E-02	9.45E-03
Freshwater ecotoxicity (CTU)	7.04E+00	4.63E+00
Freshwater eutrophication (kg P eq)	6.70E-04	5.80E-04
Marine eutrophication (kg N eq)	3.18E-03	2.72E-03
Terrestrial eutrophication (mol N eq)	2.66E-02	2.17E-02
Carcinogenic effects (ctuh)	2.99E-07	9.49E-08
Ionising radiation (kg U235 eq)	1.40E-01	1.21E-01
Non-carcinogenic effects (ctuh)	3.35E-07	2.39E-07
Ozone layer depletion (kg CFC-11 eq)	3.12E-07	2.98E-07
Photochemical ozone creation (kg NMVOC eq)	8.14E-03	6.68E-03
Respiratory inorganics effects (disease inc)	1.32E-07	1.00E-07
Dissipated water (m3 water eq)	1.22E+00	1.09E+00
Fossil use (MJ)	3.57E+01	3.10E+01
Land use (pts)	2.01E+01	1.90E+01
Mineral/metal use (kg Sb eq)	2.64E-05	1.99E-05

## Discussion

This is the first study to quantify the sustainability of different methods of delivering FV. This study used life cycle assessment to measure the environmental impact. With any LCA, there are a number of assumptions that are made in order to generate the final values. This included assumptions made regarding the actual amount of FV used, the distance and method of staff travel to the schools and the equipment needed. The model for FV in schools was based on two existing UK services but would vary for other similar services, depending on geographical locations and logistics of the programme. These assumptions were felt to be relevant or applicable to most UK services. However, anyone seeking to use this information should look at the assumptions to be sure they apply to their own service. Another assumption in all the models was that there was no waste varnish, with the exact amount of FV applied and every drop of FV used within the tube. Another assumption was that all children were reached in a single school visit. In reality, this process is likely to be less efficient.

In Table 3, a traffic light colour system was applied to three different FV scenarios: 1) FV in school; 2) FV during an existing practice appointment; and 3) FV at a separate practice appointment. School-based varnish programmes have a number of different inputs compared with practice-based appointments. However, the point of this comparison is to allow decision-makers to understand the differences in environmental impact resulting from each type of intervention. FV placed during an existing practice appointment was understandably the lowest impact way to deliver FV because it uses very little additional resource if the child is already attending the practice, for example, for routine recall. The problem with relying on this method of FV delivery, of course, is that this scenario would only apply to children who regularly attend dental practice and so would not reach children who struggle to access routine dental care.

Although this is a sustainability in dentistry paper, DALYs have also been reported, that are calculated from the human health environmental impact categories. The figure should be regarded with some wariness with all the assumptions associated with its calculation. As well as the assumptions that are made on the process of FV, there is also potential errors within the assumptions made in the LCA database and then further assumptions associated with the conversion of environmental inputs into human health effects. Nevertheless, the authors felt that it is worth publishing these figures as they are useful to compare the human health effects of clinical interventions, as well as being a useful way of aggregating and communicating the health impact.

The results of this study showed that applying FV at an existing appointment and/or FV programmes within communities at higher risk of caries (for example, areas of deprivation) should be prioritised over care with higher environmental footprints (for example, appointments solely for FV).

These results have implications both for the individual dental practitioner and the commissioner of dentistry in general practice and community or public health services, as well as similar programmes within the UK and internationally.

For individual dentists and dental care professionals, prioritisation should be given to ensuring that FV is placed in combination with a visit for other management, for example, routine recall or a treatment visit. Assuming staff are already wearing PPE and have at least an examination kit open, there are minimal additional resources needed (just the FV itself and cotton rolls, a Dappens pot and a microbrush). The additional time needed (an extra two minutes were allocated in this study) should be built routinely into appointments.

For commissioners of general or community dental programmes, this study supports FV in schools over delivery in dental practice at additional appointments. There are ways to improve the environmental impact of school programme; as demonstrated in this paper; moving from disposable examination kits to reusable kits resulted in a modest improvement in environmental emissions. In general, across all of the scenarios explored, staff and patient travel was a major contributor to the overall environmental impact. A proportion of staff travel was allocated for each FV application and patient travel for application in a dental practice. This demonstrates a wider need to increase sustainable transport in the UK. Individuals should consider how to improve their own travel to work and government/community schemes should encourage more sustainable forms of travel.

## Conclusion

In this study, applying FV in dental practice while the child is already sitting in the dental chair for another appointment was the most sustainable method of delivery, followed by FV delivery in schools. This study does not support bringing children into dental practice for an appointment solely for FV application.

For discussion of how FV application compares with other preventive programmes, the reader is invited to read the following two papers within this series that will look at toothbrushing programmes and water fluoridation.

## Supplementary Information

Supplementary Information (PDF 89KB)
