# Supplementary material for: The environmental impact of community caries prevention - part 1: fluoride varnish application
Source: Br Dent J. 2022 Aug 26;233(4):287–94. doi: 10.1038/s41415-022-4901-7 (PMC9417978; doi:10.1038/s41415-022-4901-7)
Supplement: Supplementary file 1 — Supplementary Information (PDF 89KB) [file 41415_2022_4901_MOESM1_ESM.pdf]

Online supplementary material

Life cycle inventories used in this study

Description	Amount	Unit	Ecoinvent provider
Fluoride varnish application in school			
Fluoride varnish	0.05	Number of tubes	Authors own FV tube process (see below)
FV disposables kit (2 cotton rolls, 1 dappens pot, microbrush)	2	Number of sets	Authors own FV kit process (see below)
Pulp tray	2	Number of items	Authors own pulp tray process (see below)
Disposable examination kit	2	Number of items	Process taken from Bryne et al 2021[19]
Visor with removable shield	2	Number of items	Process taken from Almutairi et al 2021[20]
Surgical mask	2	Number of items	Process taken from Almutairi et al 2021[20]
Plastic apron	2	Number of items	Authors own plastic apron process (see below)
Pair of nonsterile gloves	2	Number of pairs	Process taken from Hasan et al 2021[21]
Staff round trip from dental centre to school	0.24444	km	transport, passenger car, EURO 5 | transport, passenger car, EURO 5 | Cutoff, U - RER
Staff travel to and from dental centre	0.44444	Number of round trips	Authors own staff travel process (see below)
At an existing dental practice appointment			

Fluoride varnish	0.05	Number of tubes	Authors own FV tube process (see below)
FV disposables kit (2 cotton rolls, 1 dappens pot, microbrush)	2	Number of sets	Authors own FV kit process (see below)
Staff travel to dental practice	0.0444	Number of round trips	Authors own staff travel process (see below)
At separate dental practice appointment			
Fluoride varnish	0.05	Number of tubes	Authors own FV tube process (see below)
FV disposables kit (2 cotton rolls, 1 dappens pot, microbrush)	2	Number of sets	Authors own FV kit process (see below)
Reusable examination kit	2	Number of items	Process taken from Bryne et al 2021[19]
Visor with removable shield	2	Number of items	Process taken from Almutairi et al 2021[20]
Surgical mask	2	Number of items	Process taken from Almutairi et al 2021[20]
Plastic apron	2	Number of items	Authors own plastic apron process (see below)

Pair of nonsterile gloves	2	Number of pairs	Process taken from Hasan et al 2021[21]
Patient travel to dental practice	2	Number of round trips	Authors own patient travel to dental practice process (see below)
Staff travel to dental practice		Number of single trips	Authors own staff travel to work process (see below)

1 tube of fluoride varnish			
Ingredient: sodium fluoride	0.1808	g	market for sodium fluoride | sodium fluoride | Cutoff, U - GLO
Ingredient: ethanol	7.68	g	market for ethanol, without water, in 99.7% solution state, from ethylene | ethanol, without water, in 99.7% solution state, from ethylene | Cutoff, U - RoW
Ingredient: wax	0.02855	g	market for petroleum slack wax | petroleum slack wax | Cutoff, U - GLO
Ingredient: shellac	0.02855	g	market for epoxy resin, liquid | epoxy resin, liquid | Cutoff, U - RoW
Ingredient: colophony	0.02855	g	market for rosin size, for paper production | rosin size, for paper production | Cutoff, U - RoW
Ingredient: saccharin	0.02855	g	market for pentaerythritol | pentaerythritol | Cutoff, U - GLO
Ingredient: isoamyl acetate	0.005	g	market for 3-methyl-1-butyl acetate | 3-methyl-1-butyl acetate | Cutoff, U - GLO
Ingredient: propylene glycol	0.005	g	market for propylene glycol, liquid | propylene glycol, liquid | Cutoff, U - RoW
Ingredient: ethyl butyrate	0.005	g	market for ethyl acetate | ethyl acetate | Cutoff, U - GLO
Ingredient: geraniol	0.005	g	market for dimethyl ether | dimethyl ether | Cutoff, U - RoW
Ingredient: vanillin	0.005	g	market for vanilla | vanilla | Cutoff, U - GLO
Manufacture: electricity needed to mix FV	0.000015	kWh	market for electricity, medium voltage | electricity, medium voltage | Cutoff, U - DE

Packaging: plastic material for lid of FV tube	0.43	g	market for polypropylene, granulate | polypropylene, granulate | Cutoff, U - GLO
Packaging: injection moulding plastic lid of FV tube	0.43	g	injection moulding | injection moulding | Cutoff, U - RER
Packaging: material and manufacture of aluminium FV tube	3.51	g	market for impact extrusion of aluminium, 1 stroke | impact extrusion of aluminium, 1 stroke | Cutoff, U - GLO
Packaging: offset printing on packaging	3.51	g	offset printing, per kg printed paper | printed paper, offset | Cutoff, U - RoW
Packaging: electricity needed to fill and seal packaging tubes	0.00138889	kWh	market for electricity, medium voltage | electricity, medium voltage | Cutoff, U - DE
Packaging: electricity needed for tube capping machine to cap the FV tube	0.00357143	kWh	market for electricity, medium voltage | electricity, medium voltage | Cutoff, U - DE
Packaging: cardboard box	7.75	g	market for carton board box production, with offset printing | carton board box production, with offset printing | Cutoff, U - GLO
Transport: land (lorry)	0.874236	kg*km	transport, freight, lorry, all sizes, EURO6 to generic market for transport, freight, lorry, unspecified | transport, freight, lorry, unspecified | Cutoff, U - RoW
Transport: ferry	35.8824653	kg*km	transport, freight, sea, container ship | transport, freight, sea, container ship | Cutoff, U - GLO
Disposal: used FV tube in clinical waste	3.94	kg	market for hazardous waste, for incineration | hazardous waste, for incineration | Cutoff, U - Europe without Switzerland
Disposal: packaging in recycling	7.75	kg	market for waste paperboard | waste paperboard | Cutoff, U - GB
FV kit (2 cotton wool rolls, 1 dappens pot, 1 microbrush)			
Material: cotton wool rolls x 2	1.52	g	market for fibre, cotton | fibre, cotton | Cutoff, U - GLO
Material: polypropylene for dappens pot	1.59	g	market for polypropylene, granulate | polypropylene, granulate | Cutoff, U - GLO
Material: polypropylene for microbrush	0.58	g	polypropylene production, granulate | polypropylene, granulate | Cutoff, U - RER

Manufacture: injection moulding of dappens pot	1.59	g	injection moulding | injection moulding | Cutoff, U - RER
Manufacture: injection moulding of microbrush	0.58	g	injection moulding | injection moulding | Cutoff, U - RER
Packaging: cotton roll plastic film wrap (10x pk 50)	0.0462	g	market for packaging film, low density polyethylene | packaging film, low density polyethylene | Cutoff, U - GLO
Packaging: dappens pot plastic film wrap (pk 50)	0.0252	g	market for packaging film, low density polyethylene | packaging film, low density polyethylene | Cutoff, U - GLO
Packaging: injection moulding of microbrush pot (pk 100)	0.12	g	injection moulding | injection moulding | Cutoff, U - RER
Packaging: polypropylene for microbrush pot	0.12	g	market for polypropylene, granulate | polypropylene, granulate | Cutoff, U - GLO
Transport: land (lorry)	1.08562758	kg*km	transport, freight, lorry, all sizes, EURO6 to generic market for transport, freight, lorry, unspecified | transport, freight, lorry, unspecified | Cutoff, U - RER
Transport: sea (ferry)	0.8461452	kg*km	transport, freight, sea, ferry | transport, freight, sea, ferry | Cutoff, U - GLO
Disposal: cotton roll packaging in recycling	0.0462	g	market for waste polyethylene | waste polyethylene | Cutoff, U - GB
Disposal: cotton wool rolls x 2 in clinical waste	1.52	g	market for hazardous waste, for incineration | hazardous waste, for incineration | Cutoff, U - Europe without Switzerland
Disposal: dappens pot in clinical waste	1.59	g	market for hazardous waste, for incineration | hazardous waste, for incineration | Cutoff, U - Europe without Switzerland
Disposal: dappens pot packaging in recycling	0.0252	g	market for waste polyethylene | waste polyethylene | Cutoff, U - GB
Disposal: microbrush in clinical waste	0.58	g	market for hazardous waste, for incineration | hazardous waste, for incineration | Cutoff, U - Europe without Switzerland
Disposal: microbrush pot in recycling	0.12	g	market for waste polyethylene | waste polyethylene | Cutoff, U - GB
1 pulp tray			
Material: pulp	21.88	g	market for chemi-thermomechanical pulp GLO
Manufacture: pressing tray shape	0.0294	kwh	market for electricity, medium voltage GB

Packaging: cardboard box (pk 300)	1.2	g	market for carton board box production with offset printing GLO
Transport: land (lorry)	6.455476	kg*km	transport, freight, lorry, EUR06
Transport: sea (lorry)	5.03144	kg*km	transport, freight, sea, ferry
Disposal: packaging in recycling	1.2	g	market for waste paperboard GB
Disposal: pulp tray in clinical waste	21.88	g	Market for hazardous waste, for incineration (Europe without Switzerland)
1 plastic apron			
Material: polyethylene	15.66	g	market for polyethylene, high density, granulate GLO
Manufacture: extrusion in sheets	15.66	g	extrusion, plastic film RER
Packaging: plastic film wrap (pk 100)	0.128	g	market for packaging film, low density polyethylene
Packaging: sealing/rolling packs	4.1667E-06	kWh	market for electricity, medium voltage GB
Transport: land (lorry)	4.4159036	kg*km	transport, freight, lorry, EUR06
Transport: sea (lorry)	3.441784	kg*km	transport, freight, sea, ferry
Disposal: packaging in recycling	0.128	g	market for waste polyethylene GB
Disposal: apron in clinical waste	15.66	g	Market for hazardous waste, for incineration (Europe without Switzerland)
1 staff travel to work (round trip)			
Car	28408.5083	m	transport, passenger car, EURO 5 | transport, passenger car, EURO 5 | Cutoff, U - RER
Train	7.74757045	p*km	transport, passenger train | transport, passenger train | Cutoff, U - RoW
Bicycle	0.56772899	p*km	transport, passenger, bicycle | transport, passenger, bicycle | Cutoff, U - RoW
Scooter/motorcycle	0.28929676	p*km	transport, passenger, motor scooter | transport, passenger, motor scooter | Cutoff, U - RoW
Bus	2.32342585	p*km	transport, regular bus | transport, regular bus | Cutoff, U - RoW
Tram	2.15531673	p*km	transport, trolleybus | transport, trolleybus | Cutoff, U - RoW
1 patient travel to dental practice (round trip)			
Car	16480.9932	m	transport, passenger car, EURO 5 | transport, passenger car, EURO 5 | Cutoff, U - RER

Train	4.49469765	p*km	transport, passenger train | transport, passenger train | Cutoff, U - RoW
Bicycle	0.32936392	p*km	transport, passenger, bicycle | transport, passenger, bicycle | Cutoff, U - RoW
Scooter/motorcycle	0.16783345	p*km	transport, passenger, motor scooter | transport, passenger, motor scooter | Cutoff, U - RoW
Bus	1.34791891	p*km	transport, regular bus | transport, regular bus | Cutoff, U - RoW
Tram	1.25039161	p*km	transport, trolleybus | transport, trolleybus | Cutoff, U - RoW
